# The dural tail in intracranial meningioma: Heads up or tail down? A systematic review of the literature

**DOI:** 10.1007/s10143-025-03658-z

**Published:** 2025-06-21

**Authors:** Q. C. F. Cordia, B. M. Dijkstra, R. J. M. Groen

**Affiliations:** 1 Department of Neurosurgery, University Medical Centre Groningen, Hanzeplein 1, P.O. Box 30.001, Groningen, 9700RB The Netherlands; 2 Department of Neurosurgery, Maastricht UMC+, Maastricht, The Netherlands; 3 Department of Neurosurgery, Faculty of Medicine, Universitas Airlangga, Dr. Soetomo General Academic Hospital, Surabaya, Indonesia

**Keywords:** Meningioma, Dural tail, Histopathology, Magnetic resonance imaging

## Abstract

Meningiomas are the most common primary intracranial tumours. Recurrence occurs in up to 20%, with even higher percentages in atypical or malignant meningiomas. The non-removal of the dural tail may be a risk factor for recurrence. In this review, we aimed to determine the prevalence of tumour tissue in the radiological dural tail sign. Our secondary outcome was to determine the clinical relevance of the dural tail, if possible. PubMed, Embase, ISI Web of Science and the Cochrane Library were systematically searched for studies. Articles evaluating dural tails with both radiological and histopathological findings were included. Two reviewers independently screened studies. Data was extracted manually. 18 articles met the inclusion criteria out of 843 identified reports. In total, 406 patients with a radiological and histopathological evaluated dural tail were found. 286 patients (70.9%) had tumour infiltrate; 110 non-neoplastic dural tails showed vascular changes, loose connective tissue proliferation or inflammation. 65 patients with seemingly normal dura mater were reported, of which 26 (40.0%) showed tumour infiltration. It appears that tumour tissue in the radiological dural tail is common. Concerningly, approximately 40% of non-enhancing dura also revealed tumour tissue in the pathological examination. This highlights the need for further systematic research into the dural tail and its clinical impact on recurrence.

**Clinical trial number** Not applicable.

**Supplementary Information:**

The online version contains supplementary material available at 10.1007/s10143-025-03658-z.

## Introduction

Meningiomas are the most common primary intracranial tumours in adults, with an incidence of up to 10 per 100,000 individuals [1]. In approximately half of these cases, the diagnosis is made based on coincidental finding on radiological imaging for unrelated symptoms. Either radiological follow-up or surgical removal is chosen depending on the severity of the symptoms. The goal of surgical treatment is most often curative, whilst preventing post-operative neurological deficits. A total of 89% of the pathological confirmed cases are benign (WHO grade I), whereas only 10% and 1% are atypical (WHO grade II) and malignant (WHO grade III), respectively [1]. Despite the high prevalence of benign meningiomas, recurrence rates are described to be 21.6% in all meningioma patients in a follow-up period of 77 months [2]. In atypical or malignant meningiomas, the percentage of recurrence is described to be as high as 59% and 94%, respectively [3].

Several factors have been associated with an increased recurrence rate in meningiomas, including WHO tumour grade and extent of resection as described by Simpson resection grade. Simpson et al. demonstrated that residual tumour tissue has a negative influence on the prognostic outcome and thus, the surgical aim should be to achieve maximal safe tumour resection [4]. The dural tail might play a role in this aim, as the location of the dural tail (and non-removal of the possible residing tumour cells) could be a site of recurrence. A more radical resection margin is believed to lead to better progression-free survival [5, 6].

Thus, identifying the dural tail prior to surgical resection is critical for surgical planning. The dural tail was first described radiologically by Wilms et al. in 1989 as dural thickening on preoperative MRI, sometimes labelled as “meningeal sign” or “flare sign” [7]. In 1990, Goldsher et al. developed radiological criteria for the dural tail sign (DTS) on gadolinium enhanced MRI images as follows: (1) presence of DTS on at least 2 consecutive sections cut through the tumour at the same site and in more than one imaging plane; (2) greatest thickness adjacent to the tumour and tapering away from it; and (3) enhancement more intense than the tumour itself [8]. The dural tail can also be visible in other intracranial lesions, but in prior research, the sensitivity of DTS for meningioma was 58,6% and the specificity 94,02% [9].

Although the DTS was suggested to be a sign of tumour infiltrate in these early studies, histopathological analyses of the dura mater identified on MRI as DTS revealed the possibility of proliferation of connective tissue and/or hypervascularity [10, 11]. This called into question the aetiology of the DTS. In this study, we reviewed literature regarding the radiological imaging in relation to the pathological meningioma infiltration. The primary aim of this systematic review was to assess the prevalence of tumour tissue in the dural tail, as visible on imaging to determine its relevance for surgical planning. Our secondary aim was to critically appraise the current literature.

## Materials and methods

A literature search was performed in PubMed, Embase and ISI Web of Science. Additionally, the Cochrane Library (2024, issue 5) was searched for existing systematic reviews. Search terms included (synonyms of) (a) meningioma and (b) dural tail. Full search strategies for each database are presented in Supplementary Table S1 (final search January 31st, 2025). Reference lists were checked for additional relevant articles. The literature search was conducted according to the Preferred Reporting Items of Systematic Reviews and Meta-Analyses (PRISMA) statement [12]. The inclusion criteria were reports regarding the histopathological proven dural tail in intracranial meningioma in correlation to radiological findings, published in English, without restrictions regarding publication date. The articles were reviewed separately by two researchers (QC and BD). Disagreements were solved by discussion, optionally with the consultation of the senior author (RG).

The quality of each selected article was assessed using the Methodological Index for Non-Randomized Studies (MINORS) scale by two independent researchers (QC and BD) [13]. Discrepancies were solved by discussion or by consultation of the senior author (RG). This scale assessed several items regarding the scientific value of an article, such as the aim, endpoints, bias and follow-up. Depending on the study design (comparative or non-comparative), a maximum score of 16 and 24 could be given, respectively. Study reports with a score of 60% of the maximum score were categorized as sufficient quality studies, whereas those with a score lower than 60% were considered poor quality studies [13]. The quality assessment scores were not used to determine which article were included or excluded.

Data was manually extracted using an extraction sheet (Microsoft Excel, 2021), designed for the purpose of this study. Study characteristics included study design (prospective/retrospective), number of participants, country of origin and inclusion/exclusion criteria. Patient characteristics were described with age, gender, and tumour localisation. Radiological data comprised imaging, sequences and visibility of radiological dural tail sign. Pathological data included WHO grade, subtype, staining, dural invasion and grading of the invasion. If data was not available as described on the extraction sheet, we manually calculated the variable to fit our formatting. All data were presented descriptively in tables.

## Results

### Study selection

**Fig. 1:**
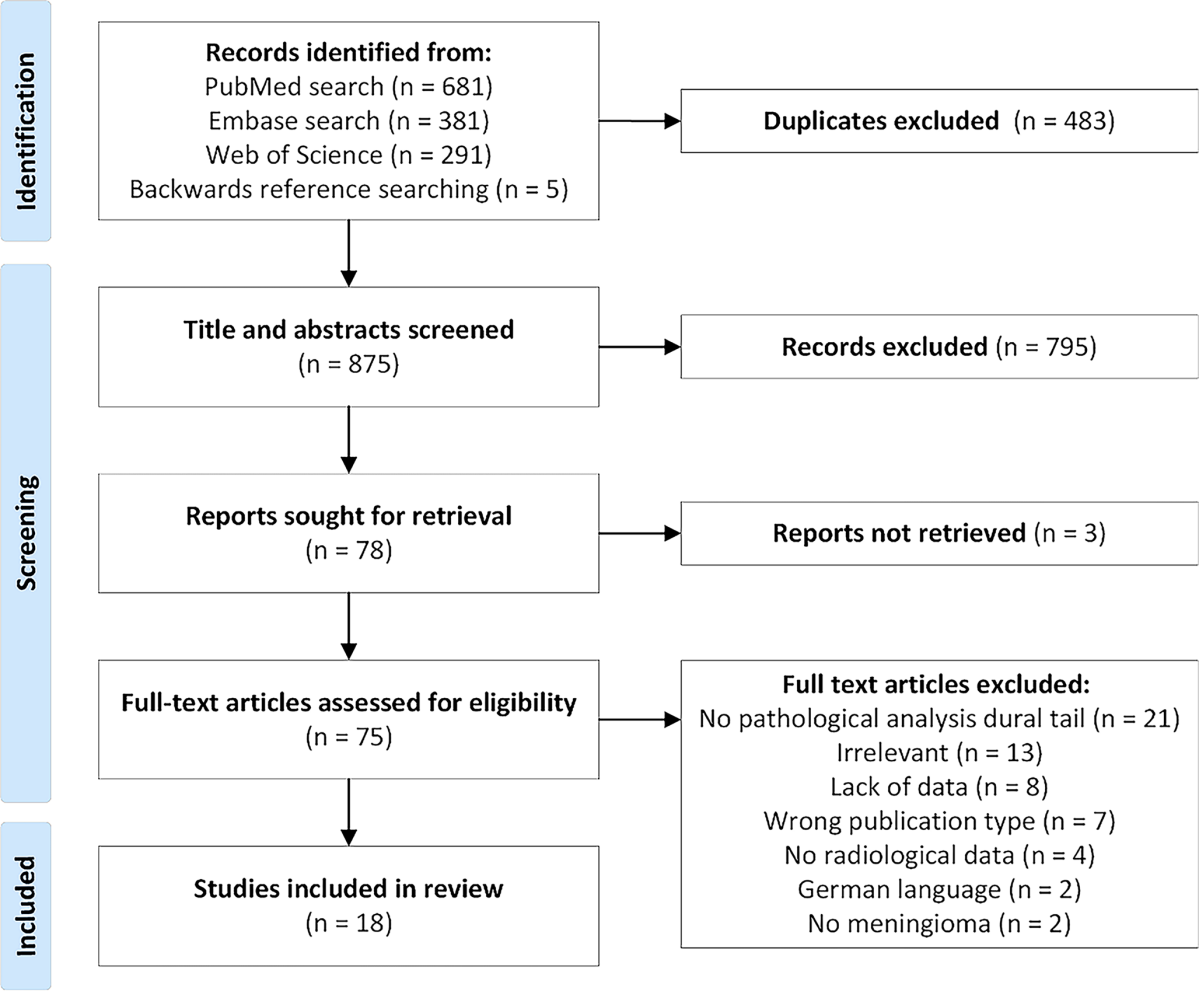
PRISMA flowchart for the study selection process. Final search performed 31st January, 2025. PRISMA Preferred Reporting Items for Systematic reviews and Meta-Analyses

In total, the search strategy and backward reference searching yielded 1358 papers. An additional five papers were identified by reference screening. After duplicate removal, 875 abstracts were identified and screened. A total of 80 articles remained for further evaluation. Three of those reports could not be retrieved and finally, 18 reports met all inclusion criteria (see PRISMA flow diagram in Fig. 1).

### Study characteristics

In this review, 12 retrospective and 6 prospective studies were included (Table 1). Articles were published between 1989 and 2021. The majority of the included studies were performed in Western countries (9/18), followed by Asian countries (8/18). One study was performed in Iran. The total number of patients described in these studies was 588. Some articles reported a discrepancy between patients with both radiological and pathological data, thus the total number of patients with both available is 432. The quality as assessed by the MINORS scale ranged from 6 to 75%. Five out of the 18 studies scored higher than 60% (Supplementary Figure S2).

**Table 1:** Study characteristics in chronological order. Total number of patients with both radiological and pathological data available is marked in grey

Study	Publication year	Country	Study design	MINORS score	*N*_total_	*N*_radio_	*N*_patho_
Wilms [7]	1989	Belgium	Retro	6	7	7	3
Aoki [27]	1990	Japan	Retro	38	18	18	3
Tokumara [17]	1990	Japan	Retro	25	4		
Goldsher [8]	1990	USA	Retro	56	30	30	5
Larson [25]	1992	USA	Retro	13	2		
Ahmadi [18]	1994	USA	Pros	44	29		
Nägele [19]	1994	Germany	Pros	25	17	17	4
Nakau [20]	1997	Japan	Retro	38	56	44	9
Hutzelmann [21]	1998	Germany	Retro	25	54		
Kawahara [22]	2001	Japan	Retro	31	7		
Takeguchi [23]	2004	Japan	Retro	38	48	48	5
Bassiouni [26]	2006	Germany	Retro	69	16	15	16
Rokni-Yazdi [14]	2009	Iran	Pros	75	16	16	12
Qi [24]	2012	China	Retro	56	179		
Slot [16]	2013	Netherlands	Pros	56	11		
Wen [11]	2014	South Korea	Retro	63	54		
You [10]	2016	China	Pros	63	18		
Kalasauskas [15]	2021	Germany	Pros	69	22	18	22

MINORS Methodological Index for Non-Randomized Studies. N_patho_ Number of patients with pathological examination of the dural tail available. N_radio_ Number of patients with radiological data of the dural tail available. N_total_ Total patients included in the original study. Pros Prospective. Retro Retrospective. USA United States of America

### Patient characteristics

The mean age ranged from 38 to 62 years old (Table 2). Five studies did not report age. Female predominance was seen in almost all studies reporting gender distribution. In 10 reports, the majority of the patients had a WHO grade I meningioma, while the other studies did not report WHO grade. Notably, the WHO classification version was not reported in half of the studies. The most frequently reported extent of resection was Simpson grade I (ranging from 88 to 100% in nine studies).

**Table 2:** Patient characteristics of all included patients

Study	*N*_total_	Age (mean (range))	Sex (% female)	WHO grade (%)	Simpson resection grade (%)
Wilms [7]	7	ND	ND	ND	ND
Aoki [27]	18	53 (27–72)	67	ND	ND
Tokumara [17]	4	55 (50–58)	100	WHO I (100)	Grade I (100)
Goldsher [8]	30	59 (ND)	ND	ND	ND
Larson [25]	2	38 (20–55)	50	WHO I (100)	Grade I (100)
Ahmadi [18]	29	ND	ND	ND	ND
Nägele [19]	17	ND	ND	ND	ND
Nakau [20]	56	62 (41–84)	56	WHO I (100)	ND
Hutzelmann [21]	54	ND	ND	ND	Grade I (100)
Kawahara [22]	7	55 (39–70)	71	WHO I (100)	Grade I (100)
Takeguchi [23]	48	54 (26–77)	72	ND	ND
Bassiouni [26]	16	55 (31–80)	63	WHO I (88) WHO III (6)	Grade I (88) Grade III (12)
Rokni-Yazdi [14]	16	ND	ND	ND	ND
Qi [24]	179	59 (15–81)	71	WHO I (86) WHO II (7) WHO III (7)	Grade I (100)
Slot [16]	11	53 (39–71)	45	WHO I (73) WHO II (27)	Grade I (100)
Wen [11]	54	57 (ND)	52	WHO I (75) WHO II (25)	ND
You [10]	18	57 (22–80)	76	WHO I (100)	Grade I (100)
Kalasauskas [15]	22	54 (30–85)	91	WHO I (73) WHO II (27)	Grade I (91) Grade II (10)

ND Not described. N_total_ total patients. WHO World Health Organization

### Tumour infiltration of the dural tail

**Fig. 2:**
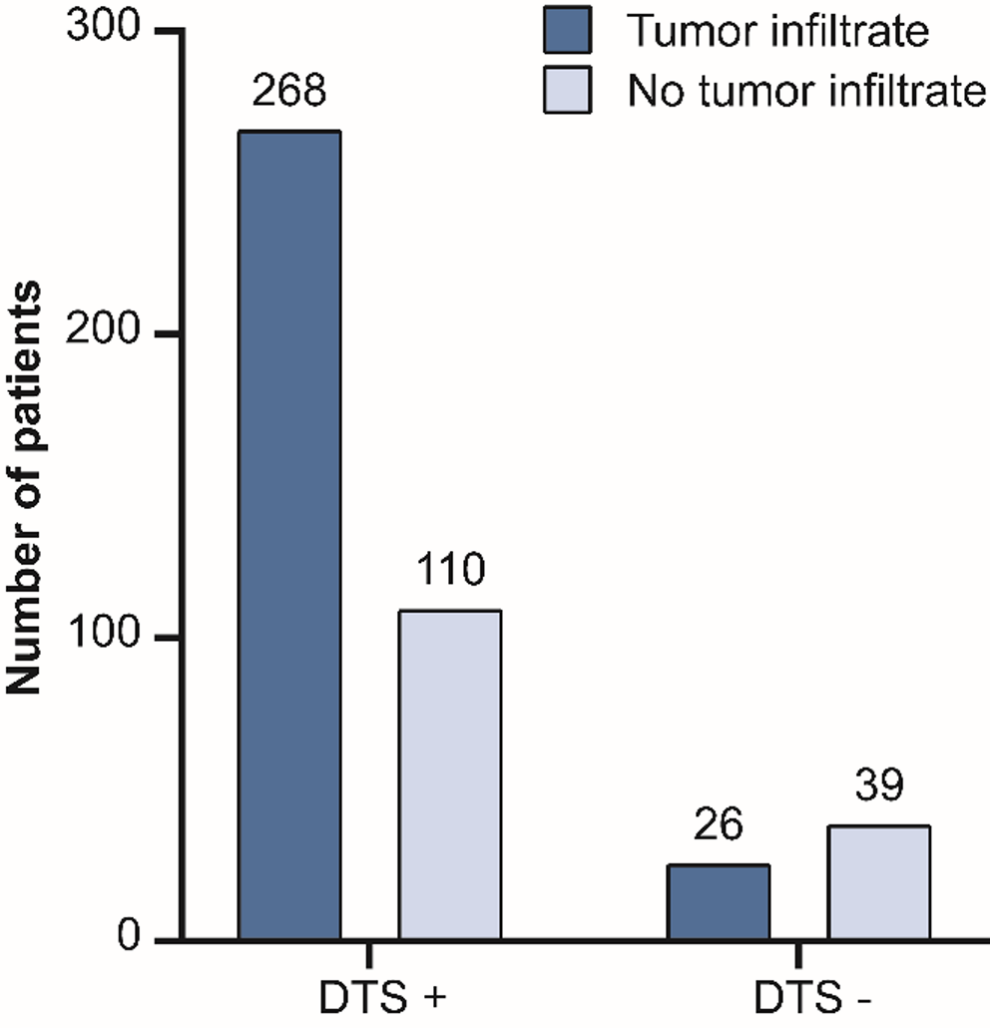
Graphical representation of number of patients with dural tail sign and tumour infiltrate. DTS dural tail sign

The percentage of pathological proven tumour invasion in the radiological DTS varied from 0 to 100%, in studies reporting 2 to 179 patients (Table 3). In total, 378 patients were reported with a positive radiological DTS. The dural tail contained histopathological confirmed tumour infiltrate in 268 patients (70.9%) (Fig. 2). Some authors tried to correlate the infiltration to clinical variables, such as age, sex, pattern of enhancement, size of the primary lesion or the size of the dural tail. Rokni-Yazdi et al., Kalauskas et al. and Wen et al. found no statistically significant relationship [11, 14, 15]. Wen et al. did find that malignant meningiomas showed enhanced dura more often than benign meningiomas (*p* = 0.02) [11]. Slot et al. found that all WHO grade II patients had tumour infiltrate in the dural tail but described no statistical relation [16].

**Table 3:** Pathological proven tumour invasion in radiological dural tail sign

Study	*N*_total_	*N*_radio + patho_	DTS+	DTS + TI+	% TI + in DTS+	DTS-	DTS- TI+	% TI + in DTS-
Wilms [7]	25	3	3	3	**100**	ND		
Aoki [27]	17	3	3	0	**0**	ND		
Tokumara [17]	7	4	4	2	**50**	ND		
Goldsher [8]	16	5	3	3	**100**	2	2	**100**
Larson [25]	179	2	2	2	**100**	ND		
Ahmadi [18]	29	29	22	16	**72.7**	7	2	**28.6**
Nägele [19]	16	4	4	0	**0**	ND		
Nakau [20]	11	9	9	4	**44.4**	ND		
Hutzelmann [21]	30	54	31	20	**64.5**	23	7	**30.4**
Kawahara [22]	56	7	7	1	**14.3**	ND		
Takeguchi [23]	54	5	5	4	**80**	ND		
Bassiouni [26]	18	15	11	2	**18.2**	4	3	**75.0**
Rokni-Yazdi [14]	4	12	12	5	**41.7**	ND		
Qi [24]	48	179	179	158	**88.3**	ND		
Slot [16]	22	11	11	6	**54.5**	11*	4	**36.4**
Wen [11]	7	54	36	22	**61.1**	18	8	**44.4**
You 10]	18	18	18	17	**94.4**	ND		
Kalasauskas [15]	68	18	18	3	**16.7**	ND		

DTS + Radiological dural tail sign present. DTS- Radiological dural tail sign absent. N _radio + patho_ Number of patients with both radiological and pathological data available. N _total_ Total patients included. TI + Pathological tumour infiltration found. TI- No pathological tumour infiltration found

* Reported on non-enhancing dura in tumours with DTS present

On the other hand, 110 patients (29.1%) showed no tumour infiltration of the DTS. Instead, vascular changes, e.g. dilatation and proliferation [10, 11, 17, 18–14, 23, 24], loose connective tissue proliferation [17, 20–22, 24], inflamed dura [11, 14, 24], reactive hyperplasia [24], dense fibrous tissue [25] and congestion [22] were described.

Six studies also reported on the tumour infiltrate in tumours with a negative DTS [8, 11, 16, 18, 21, 26]. In 65 patients with seemingly radiological normal dura mater, 26 (40.0%) showed tumour infiltration. This was reported in 2 to 23 patients, with a range of 28.6–100%.

Some authors have tried to predict the presence or absence of tumour infiltrate. The reports of Goldsher and Nägele found no tumour infiltration and offered that the hyperintensity of the tail was an indication of a histological different tissue than the tumour [8, 19]. Takeguchi et al. reported an isointense or slightly higher signal in tumour infiltrated dural tail, whereas the non-invaded dural tail had a very high contrast-enhanced signal [23]. Nonetheless, Ahmadi and coworkers offered that the pattern of enhancement was more important than the mere presence of DTS [18]. A discontinuous DTS would represent dural invasion, whereas a continuous DTS is caused by inflammatory changes. This is in line with the work of Qi et al.: they further developed a classification of the DTS in five categories: smooth, nodular, mixed, symmetrical multipolar and asymmetrical multipolar. They found that a smooth DTS correlated with less tumour infiltrate compared to nodular DTS [24].

## Discussion

Our systematic review showed that little data is available for evaluating the histopathology of dural invasion in the radiological dural tail, based on 18 studies. The radiological DTS occurred between 34% and 72% of meningioma patients in the included reports [8, 27, 19–21, 23]. This corresponds to the data of two separate radiological imaging studies (with no pathological data), who reported that in approximately 60% of the meningiomas DTS is visible [9, 28]. Since the first identification of the radiological dural tail by Wilms et al. more than 30 years ago, only 378 histologically evaluated dural tails have been reported in the literature [7]. In 268 (70.9%) of these tails, tumour infiltration was found. Furthermore, tumour infiltration was found in 26 out of 65 patients (40.0%) where a DTS was absent.

Several hypotheses regarding the aetiology of the DTS have been proposed. Although *neoplastic* (or pathognomonic) tumour invasion was the original hypothesis of the first article describing the dural tail [7], a *non-neoplastic* pathophysiological mechanism was subsequently described in literature. It was uncovered that the dural tail sign was nonspecific for meningiomas and was also present in other intracranial masses [9, 29]. Two subsequent studies found that the dural tail had no tumoral involvement distant from the tumour mass to explain the observed dural tail [17, 27]. Hence, they offered the non-neoplastic mechanism: the DTS could be caused by reactive changes to the tumour mass resulting in hypervascularity and connective tissue changes. Kawahara et al. added to the non-neoplastic pathophysiology hypothesis, by describing tumour cells invading dural vessels and “packing” them at the point of the tumour attachment [22]. This phenomenon induced vessel congestion and consequently, the dural tail sign. Finally, Wen and colleagues speculated that local compression of the tumour mass adjacent to the dura mater induces non-neoplastic reactive dural inflammation [11]. Simultaneously, they also offered that fragile neovasculature could easily be invaded by tumour cells, which could result in neoplastic distal tumour invasion. This was later supported by results from Kalauskas’ group: 8.1% of the distal dural tail samples contained meningioma cells and in 6.5% there was no exclusion of tumour possible [15]. Nevertheless, this current review suggests that the dural tail sign on preoperative imaging is an indicator of tumour infiltration in these patients.

Regardless of this non-neoplastic hypothesis, this review also shows that meningioma tissue can be found in more than a third (40%) of the non-enhancing dura. This highlights the concerning need for prediction methods of tumour invasion in the dura both pre- and intra-operatively. Nevertheless, no consensus exists regarding the dural tail in clinical decision making (e.g. more radical treatment plan) or predicting patient outcomes (e.g. long-term recurrence free survival). In neuroradiotherapy, several studies have shown that radiation of the dural tail does not improve tumour recurrence rates in a follow-up period between 3,3 and 7,5 years [30–33]. On the contrary, including the dural tail in the treatment plan for radiotherapy increases the treatment volume and thus the risk of adverse effects due to collateral brain radiation. In neurosurgery, the clinical benefit of a radical resection of the dural tail has also not been proven. Ildan and colleagues described that the presence of a radiological dural tail did not affect the recurrence rate in univariate analysis, but in multivariate analysis it did affect the time to recurrence [34]. Nonetheless, they did not describe the difference in time to recurrence and whether this was clinically relevant. Nakusa et al. found no statistically significant relationship between the dural tail and recurrence, but they described that meningiomas with a dural tail recurred in the area or border of the dural tail [35]. Furthermore, Ong and coworkers have found that approximately two thirds of meningiomas recur within the resection cavity and one third outside of it but did not specify whether these patients had a dural tail or whether this was resected [36]. In light of this conflicting evidence, some groups have investigated the feasibility of extending the dural resection margin by means of grade 0 resection or fluorescence guided surgery, but the clinical impact remains to be investigated [24, 37–40].

This lack of evidence highlights the major issue in meningioma dural tail research: the paucity of the evaluated dural tails compared to the total incidence of meningiomas. The majority of included studies had a low level of evidence, as scored by the MINORS scale. Most reports were outdated (> 15 years old since current report), when common practice was significantly different from both a clinical and a research viewpoint. Almost half of the studies reported on less than 10 dural tails with both radiological and pathological data. Furthermore, the radiological criteria were well-defined based on Goldsher in the minority of articles (8/18) [8]. Others only referred to these criteria (4/18) or adapted a very similar definition (4/18). Three reports did not provide a description of the radiological DTS, of which two reports were published prior to Goldsher’s work [7, 17]. These issues make it extremely difficult to evaluate clinical and statistical significance and correlate the dural tail to clinical variables or recurrence. Most studies had a discrepancy between the number of patients with radiological and histopathological examination. This raise concerns regarding selection bias. Furthermore, approximately half of the reports lack essential patient data, such as WHO grade and extent of resection. This highlights the need for systematic radiological and histopathological evaluation of the dural attachment of meningiomas to further elucidate the role of the dural tail and dural invasion in the risk of recurrence. Although this review mainly focusses on intracranial meningioma, similar findings have been reported for spinal meningiomas: in 65 spinal meningioma patients, 28 patients (43.0%) showed invasion of the adjacent dura mater on the radiological dural tail sign [41, 42]. The high prevalence of tumour in dural tails in meningiomas is striking. However, due to the low quantity and poor quality of the available literature, statistical analysis in this review is not possible. As such, no recommendations for the development of new guidelines or evaluation of existing guidelines can be made at present.

### Future perspectives

In conclusion, with only 18 included articles, the results of this review highlight that substantial research on the dural tail is warranted. This study suggests that the majority of the dural tail sign on preoperative imaging is as a result of tumour infiltrate. Furthermore, more than one third of non-enhancing dura in this literature shows tumour infiltrate. More large-scale prospective studies with systematic reporting, including the dural tail and its histopathological evaluation as outcome measure, are needed to confirm this finding, as current literature is outdated and qualitatively lacking. Intra-operative imaging methods, such as tumour cell fluorescence imaging, might be helpful to identify meningioma in the enhancing and non-enhancing dura mater. Additionally, more research is needed to elucidate the effect of the (non-) removal of the dural tail on tumour recurrences and patient reported outcomes. The identification of the clinical relevance of the dural tail will help to develop and tailor evidence-based guidelines for the treatment of intracranial meningiomas.

## Electronic supplementary material

Below is the link to the electronic supplementary material.

Supplementary Material 1

## Data Availability

No datasets were generated or analysed during the current study.

## Abbreviations

DTS: Dural Tail Sign
EoR: Extent of Resection
MINORS: Methodological Index for Non-Randomized Studies
PRISMA: Preferred Reporting Items of Systematic Reviews and Meta-Analyses
WHO: World Health Organization
TI: Tumour Infiltration
MRI: Magnetic Resonance Imaging
