# Supplementary material for: The dural tail in intracranial meningioma: Heads up or tail down? A systematic review of the literature
Source: Neurosurg Rev. 2025 Jun 21;48(1):520. doi: 10.1007/s10143-025-03658-z (PMC12181092; doi:10.1007/s10143-025-03658-z)
Supplement: Supplementary file 1 — Supplementary Material 1 [file 10143_2025_3658_MOESM1_ESM.docx]

**Supplementary data**

| **PubMed** | **Embase** | **ISI Web of Science** |
| --- | --- | --- |
| (Meningioma[Mesh] OR meningioma*[tiab])  AND  ("Dura Mater/abnormalities"[Mesh] OR "Dura Mater/analysis"[Mesh] OR "Dura Mater/diagnosis"[Mesh] OR "Dura Mater/diagnostic imaging"[Mesh] OR "Dura Mater/pathology"[Mesh] OR "Dura Mater/physiopathology"[Mesh] OR dural tail*[tiab] OR flare sign*[tiab] OR tail sign*[tiab] OR dural thickening[tiab] OR DTS[tiab]) | ('meningioma'/exp OR meningioma*:ab,ti,kw)  AND  ('dural thickening’:ab,ti,kw OR ‘flare sign*’:ab,ti,kw OR ‘tail sign*’:ab,ti,kw OR DTS:ab,ti,kw OR ‘dural tail*’:ab,ti,kw) | TS = meningioma*  AND  TS= (“dural thickening” OR “flare sign*” OR “tail sign*” OR DTS OR “dural tail*”) |
| **Table S1.** **Search terms for literature search in each database.** | | |


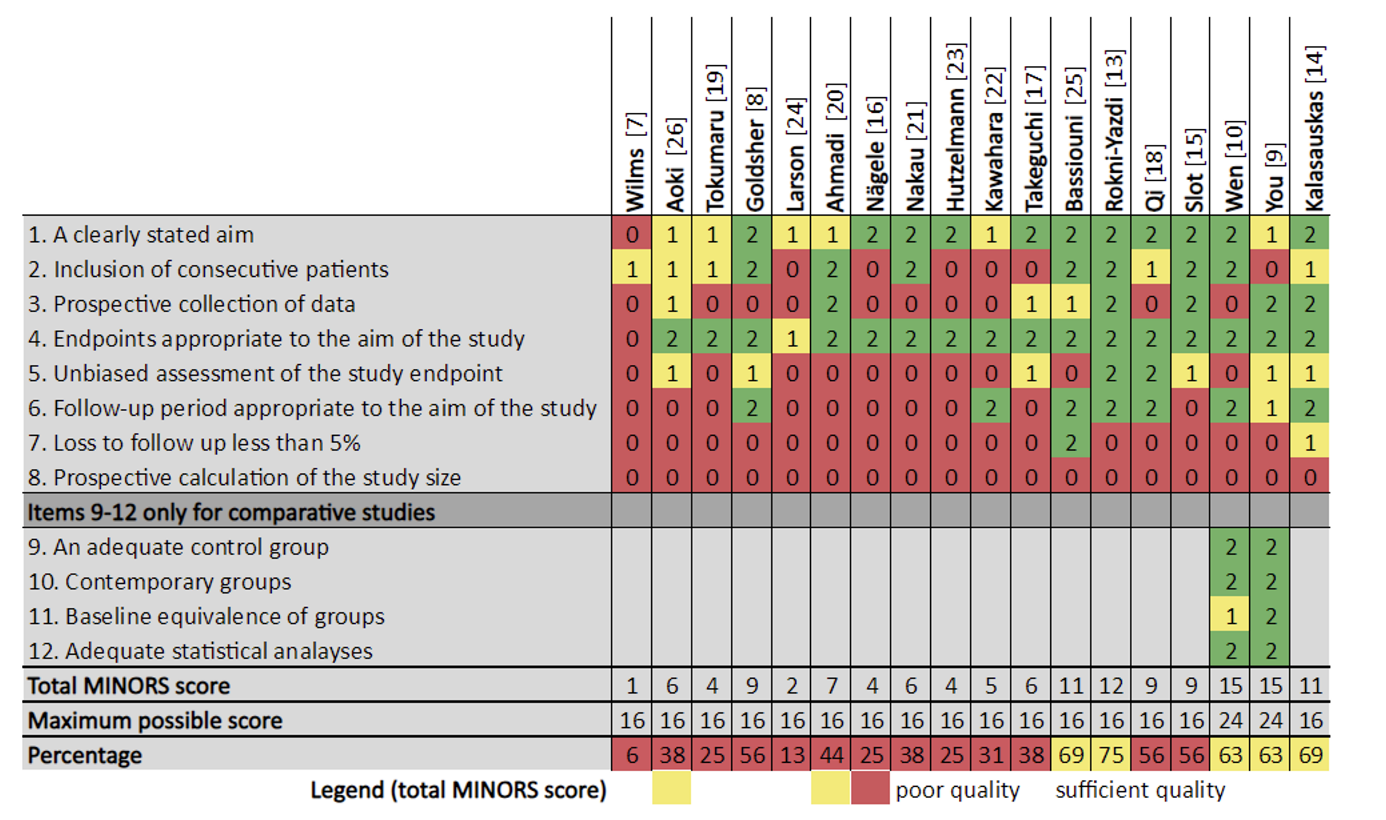


**Figure S2. MINORS quality appraisal.**
